# Is It Possible to Perform Quality Neonatal CPR While Maintaining Skin-to-Skin Contact? A Crossover Simulation Study

**DOI:** 10.3390/children11121471

**Published:** 2024-11-30

**Authors:** Myriam Santos-Folgar, Alejandra Alonso-Calvete, Adriana Seijas-Vijande, Ana Sartages-Castro, Martín Otero-Agra, María Fernández-Méndez, Roberto Barcala-Furelos, Felipe Fernández-Méndez

**Affiliations:** 1 REMOSS Research Group, Faculty of Education and Sports Sciences, University of Vigo, 36005 Pontevedra, Spain; msantos@uvigo.es (M.S.-F.); adrisei1998@gmail.com (A.S.-V.); martinoteroagra@gmail.com (M.O.-A.); mariajosefernandezmendez@gmail.com (M.F.-M.); roberto.barcala@uvigo.es (R.B.-F.); felipefernandez@uvigo.es (F.F.-M.); 2 School of Nursing, University of Vigo, 36005 Pontevedra, Spain; 3 Department of Obstetrics, Complexo Hospitalario of Pontevedra, Sergas, 36002 Pontevedra, Spain; anasartages14@gmail.com; 4 Docent Unit of Obstetrics-Gynaecology Nursing (Midwifery), Faculty of Nursing, University of Santiago de Compostela, 15782 A Coruña, Spain; 5 Faculty of Physiotherapy, University of Vigo, 36005 Pontevedra, Spain; 6 CLINURSID Research Group, Psychiatry, Radiology, Public Health, Nursing and Medicine Department, University of Santiago de Compostela, 15782 Santiago de Compostela, Spain; 7 Simulation and Intensive Care Unit of Santiago (SICRUS) Research Group, Health Research Institute of Santiago, University Hospital of Santiago de Compostela-CHUS, 15706 Santiago de Compostela, Spain; 8 Faculty of Education and Sport Sciences, University of Vigo, 36005 Pontevedra, Spain

**Keywords:** CPR: neonatal, skin-to-skin contact, delayed cord clamping, quality, emergency care

## Abstract

**Background**: This study aimed to assess the feasibility and quality of resuscitation maneuvers performed on a newborn over the mother’s body while maintaining SSC and delayed cord clamping. **Methods**: A randomized crossover manikin study compared standard cardiopulmonary resuscitation (Std-CPR) and cardiopulmonary resuscitation during SSC (SSC-CPR). Nursing students (n = 40) were recruited and trained in neonatal CPR. The quality of the CPR, including compression and ventilation variables, was evaluated using Laerdal Resusci Baby QCPR^®^ manikins. **Findings**: No significant differences were found in the compression variables between the Std-CPR and the SSC-CPR. The quality variables demonstrated comparable results between the two techniques. The quality of the compressions showed medians of 74% for the Std-CPR and 74% for the SSC-CPR (*p* = 0.79). Similarly, the quality of the ventilations displayed medians of 94% for the Std-CPR and 96% for the SSC-CPR (*p* = 0.12). The overall CPR quality exhibited medians of 75% for the Std-CPR and 82% for the SSC-CPR (*p* = 0.06). **Conclusions**: Performing CPR on a newborn over the mother’s body during SSC is feasible and does not compromise the quality of resuscitation maneuvers. This approach may offer advantages in preserving maternal–newborn bonding and optimizing newborn outcomes. Further studies are needed to address the limitations of this research, including the use of simulations that may not fully replicate real-life conditions, the lack of analysis of different types of labor, and the unpredictability of the maternal response during resuscitation.

## 1. Introduction

Most newborns adapt well to extrauterine life [1]. In these cases, immediately after birth, recommendations support placing the newborn over the mother, in skin-to-skin contact (SSC) [2,3,4,5,6,7,8], and that cord clamping should be delayed due to the demonstrated benefits versus immediate clamping, including a lower risk of anemia in the first year, higher iron and antibody levels and fewer infections [9,10,11,12,13,14,15].

However, several situations have been reported in which newborns need support and then resuscitation maneuvers are required. In these situations, the infant is placed in a thermal cradle with radiant heat in order to perform the necessary procedures, separating them from their mother after birth [1]. Thus, the newborn is deprived of the benefits of SSC and delayed cord clamping, increasing the risk of the critical situation [9,10,11,12,13,14,15]. Considering the negative effects reported when cord clamping is performed immediately and when SSC is not maintained, a recent investigation hypothesized whether it is possible to provide the best care to infants and mothers when resuscitation is required. The results of this study suggest that the evidence is limited but research in this topic is needed to provide new proposals of resuscitation that consider SSC and delayed cord clamping [16]. In accordance with this suggestion, previous studies have been conducted with study protocols analyzing the feasibility of performing CPR with delayed cord clamping while placing the infant on a table near the mother [17,18,19,20]. These investigations suggest that new protocols in pediatric CPR should be developed considering the closeness between mother and baby and late cord clamping.

However, to the best of these authors’ knowledge, no research has analyzed the feasibility of performing CPR maneuvers with the newborn over the mother’s body, preserving SSC, although the International Liaison Committee on Resuscitation (ILCOR) suggests considering whether resuscitation maneuvers can be performed during SSC in newborns [21].

Therefore, in accordance with previous research and guidelines, the purpose of this study was to analyze the feasibility and quality of resuscitation maneuvers on a newborn after birth over the mother’s body while maintaining SSC and preserving delayed cord clamping.

## 2. Materials and Methods

### 2.1. Study Design

A randomized crossover manikin study was performed to compare two simulated situations during neonatal CPR: standard CPR (Std-CPR) and skin-to-skin contact CPR (SSC-CPR). A sequence of randomized numbers was chosen in order to assign each participant to the Std-CPR group or the SSC-CPR group.

### 2.2. Participants

The subjects in this study were voluntarily recruited from the Faculty of Nursing of the University of Vigo (Pontevedra). Considering that no previous studies have compared Std-CPR with SSC-CPR or similar maneuvers, the sample size was calculated according to the protocol of Knol et al. [17], who compared CPR with immediate cord clamping with CPR with delayed cord clamping. According to the statistical results of this protocol, a sample size of 32 subjects in each group was sufficient to provide significant results, with a power of 80% and a 95% confidence interval. Finally, a sample of 40 nursing students was recruited for this crossover design in each group.

The Research Ethics Committee of the National Health Care Service (SERGAS) did not consider it necessary to review this research for being a simulation study. In addition, all subjects were informed about the procedures and their right to withdraw from this study at any moment and signed written informed consent according to the standards of the Declaration of Helsinki. The CONSORT Checklist was selected and completed for this manuscript.

### 2.3. Study Protocol

**Figure 1:**
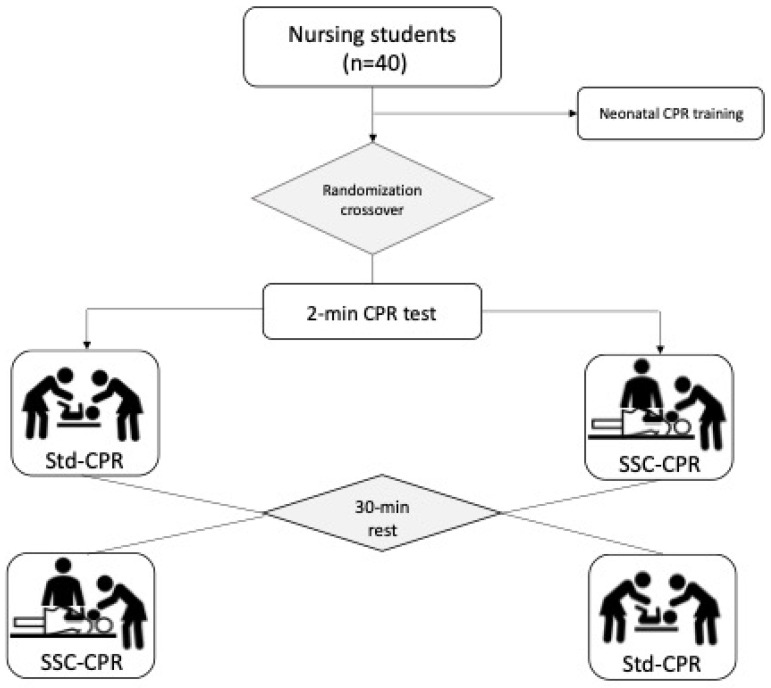
Flow chart and design. CPR: cardiopulmonary resuscitation; SSC-CPR: skin-to-skin contact cardiopulmonary resuscitation; Std-CPR: standard cardiopulmonary resuscitation.

The details are described in Figure 1.

### 2.4. Training

The participants received specific training in neonatal CPR according to their regular learning programs. This training consisted of a theoretical explanation and practical situations with simulators with a feedback system. Thus, the participants could observe, in real time, the performance of the techniques during training and improve specific skills such as ventilations or compressions. These training sessions were performed in small groups with a duration of 4 h on two different days.

### 2.5. Test

All the participants in the pairs performed (1) simulation of Std-CPR and (2) simulation of SSC-CPR. Based on recent guidelines [22], in both maneuvers, the duration was two minutes, starting with five rescue ventilations and followed by cycles of three compressions and 1 ventilation. Specifically, the compressions were performed with the two-thumb encircling technique and the ventilations with the auto-inflatable bag SPUR II Neonatal Resuscitator—300 mL (Ambu^®^ Ballerup, Copenhagen, Denmark) and a Baby Face Mask, number 0A (Ambu^®^ Ballerup, Copenhagen, Denmark). During these tests, the rescues did not change roles (compressions or ventilations) and did not receive feedback. A minimum time of rest between both tests was set up at 30 min in order to avoid fatigue.

Regarding the Std-CPR, each manikin was located on a thermal cradle (Ohmeda Ohio^®^ Infant Warmer System; Columbia, MD, 21046-1801 (A Division of The BOC Group Inc. USA)). Regarding the SSC-CPR, each manikin was placed over the body of a woman, simulating a real situation after childbirth, on a table with 45° of elevation [8]. In both conditions, the participants performed ventilations over the manikins’ heads and compressions lateral to the women and manikins.

### 2.6. Variables and Measuring Equipment

The manikin used was the Laerdal Resusci Baby QCPR^®^ (Stavanger, Norway) with the Laerdal SIMPAD register system (Stavanger, Norway). This system records variables of compression, ventilation and quality of CPR and was set up according to the recommendations of the ERC (2021) [1]. Regarding the weight of the newborn, according to the growth charts of the World Health Organization, a medium weight of 3.45 kg was calculated [23,24,25].

Concerning the ventilations, a rhythm of 30 to 60 ventilations per minute was established and 16–29 mL (5–8 mL/kg) was set as the correct volume of ventilations [1]. Regarding the compressions, a rhythm of 120–140 compressions per minute was established [1]. Regarding the depth, the ERC guidelines recommend performing the compressions with a depth of 29–33 mm [1], but the software did not allow this configuration, so a depth of 30–33 mm was established as correct [26,27].

### 2.7. CPR Variables

Chest compressions (CCs):

- Total number of CCs; mean rate in CCs/min
- Mean depth in mm
- CCs with adequate rate as a percentage
- CCs with adequate depth as a percentage
- CCs with adequate release as a percentage
- CCs with adequate hand-position as a percentage

Ventilations (Vs):

- Number of total Vs
- Effective Vs as a percentage
- Mean volume in mL

The quality parameters were assessed and disaggregated into CC quality, V quality and CPR quality. Each variable was expressed with a percentage, and its calculation was based on formulas published in previous studies [28,29,30]:

- CC quality, calculated using the following formula: (CCs with adequate depth + CCs with adequate release + CCs with adequate rate) ÷ 3;
- V quality, calculated using the following formula: number of Vs with adequate volume/number of total Vs * 100;
- CPR quality, calculated using the following formula: (CC quality + V quality)/2.

### 2.8. Statistical Analysis

Statistical analysis was performed with IBM SPSS Statistics v.20 for Windows (Armonk, NY, USA). To describe the categorical variable (sex), absolute and relative frequencies were used. The other variables were described with measures of central tendency (median: Me) and measures of dispersion (interquartile range: IQR). The normality of the distribution was checked with the Shapiro–Wilk test, and comparisons were performed with the Wilcoxon test on the variables that did not follow normal distribution and with Student’s *t*-test on the variables that followed normal distribution. Effect size (ES) was calculated with the Rosenthal test and Cohen’s d, respectively. ESs of <0.2 were trivial, ESs of 0.2–0.5 were small, ESs of 0.5–0.8 were moderate, ESs of 0.8–1.3 were large and ESs of >1.3 were very large [31]. A *p*-value of <0.05 was set up as significant.

## 3. Results

This study was carried out in 40 nursing students (87% women) with a median age of 21 years (IQR: 21–22 years), a median weight of 66 kg (IQR: 59–73 kg) and a median height of 167 cm (IQR: 163–172 cm).

### 3.1. Compression Variables

The results of the compression variables are reported in Table 1. As detailed, no significant differences were found in any of the variables between the Std-CPR and SSC-CPR, except from the mean rate (CCs/min), which had a higher number of compressions during the Std-CPR vs. the SSC-CPR (*p* = 0.004; ES = 0.46).

### 3.2. Ventilation Variables

The results for the ventilation variables are reported in Table 2. No significant differences were found in the total number of ventilations (*p* = 0.06) or the % of effective ventilations (*p* = 0.40) between the Std-CPR and SSC-CPR. Significantly less time was observed in the mean time to ventilation during the SSC-CPR vs. the Std-CPR (*p* < 0.001; ES = 0.83). The mean ventilated volume was significantly lower during the SSC-CPR in comparison with the Std-CPR (*p* = 0.042; ES = 0.45).

### 3.3. Quality Variables

The results for the quality variables during CPR are detailed in Table 3. No significant differences were found in any quality variables: neither in the quality of the compressions (*p* = 0.79) nor in the quality of the ventilations (*p* = 0.12) or the global quality (*p* = 0.06).

## 4. Discussion

This study aimed to assess the feasibility of SSC-CPR and to compare this technique with Std-CPR performed on a warm table. The main findings were that (1) performing SSC-CPR is feasible after childbirth; (2) a sample of nursing students could perform high-quality SSC-CPR and (3) there were no differences in quality between the usual CPR method (Std-CPR) and CPR with skin-to-skin contact (SSC-CPR). Thus, this investigation suggests that SSC-CPR could be performed with the same high quality as the standard techniques and with no risk, adding the benefits of maintaining skin-to-skin contact between mother and newborn.

Previous research has reported that the quality of CPR is critical to increase survival [22,23,24]. During the SSC-CPR in this study, the nursing students were trained in the method and demonstrated that they were able to perform high-quality maneuvers, with more than 70% quality, agreeing with the criteria established by Perkins et al. [32]. Moreover, no significant differences were found between the SSC-CPR and Std-CPR, so it has been suggested that maintaining the skin-to-skin contact during the resuscitation did not negatively affect the quality of the technique. In fact, slightly higher values were achieved in quality during the SSC-CPR vs. the Std-CPR (7%).

Prior studies in the same vein have also analyzed the quality of pediatric CPR performed by trained students, with similar quality values to those obtained in this study [33,34]. Nevertheless, and despite the quality of the techniques, the results of this investigation reported the need for improving the depth of the chest compressions, since they were carried out too deeply. According to the guidelines, compressions should be performed with a depth of 1/3 of the anteroposterior diameter, and recent, similar research reported no difficulties in compressing adequately on a pediatric manikin [35]. Therefore, more training might be necessary to improve this skill. However, regarding ventilation and airway management, no difficulties were observed in achieving adequate ranks of volume (20–40 mL) and the correct management of air entry with the baby over the mother with skin-to-skin contact [36]. Therefore, the results of this investigation support SSC during CPR, which may allow delayed cord clamping.

In this sense, immediate skin-to-skin contact between mother and baby has been associated with a lower risk of neonatal hypothermia and protects the baby against hyperthermia [4]. This contact also has a positive impact on cardiorespiratory stabilization [2,3], including a more effective transition to extrauterine life, with newborns presenting greater levels of cardiac frequency, respiratory frequency and oxygen saturation during the first six hours of life [37]. Additionally, other benefits of skin-to-skin contact have been described in previous studies, such as better breastfeeding, better mother–child bonding [4,5] and lower risk of hypoglycemia [6]. Although a slightly lower chest compression rate was observed in the SSC-CPR versus the Std-CPR (131 vs. 136 CCs/min, *p* = 0.004), this probably does not affect resuscitation success in stable neonates, but further studies are needed to assess its impact in more compromised neonates or in prolonged resuscitations. These compression results are in agreement with a study by Carballo-Fazanes et al. where, using a 3:1 ratio in neonatal CPR, compression rates of 133 CCs/min were achieved [35].

On the other hand, immediate umbilical cord clamping has been associated with death or neuro-disability [9,10,11,12,13] and may aggravate the situation of an already compromised newborn, especially if the cord is cut before the lungs are ventilated [38]. Thus, recent research on this topic has reported that delayed umbilical cord clamping at delivery has well-recognized benefits [39]. First, this method of clamping the umbilical cord has demonstrated a feto-placental transfusion of oxygenated venous blood, which may buffer existing acidosis. Additionally, it may increase blood volume by up to 20%, which would enhance red and white blood cells, thrombocytes, mesenchymal stem cells, immunoglobulins and iron. Due to these processes, the resulting increase in lung perfusion can compensate for existing hypoxemia or hypoxia [38]. A prior study in lambs has reported that if the umbilical cord clamping is delayed until the lungs are ventilated, there is an improvement in pulmonary blood flow, with a more stable cerebral hemodynamic transition after birth [14]. It is therefore paradoxical that immediate clamping is usually performed in neonates with asphyxia to provide immediate care and deprives them of these benefits.

Finally, regarding family–child bonding, with SSC-CPR, the presence of the family during CPR appears to be possible. The current scientific evidence supports the presence of the family during resuscitation maneuvers [1], mentioning that if resuscitation is being performed in another area, the family will be unable to see what happens to the baby, increasing the anxiety of the process [40]. Previous research by Katherina et al. described the experience of resuscitation near the bed as positive, since the family could see or touch the newborn after labor and during resuscitation. However, although the umbilical cord was intact, in this study, the CPR was not conducted skin-to-skin [41].

### Limitations and Future Perspectives

This study presents several limitations that should be considered. First, manikins were used to simulate the CPR in both the standard and skin-to-skin conditions. These results should be read carefully when extrapolating to real situations, since the performance, resources and response during a real situation could be different; in other words, the stress conditions in a real emergency are impossible to be simulated, and the response of the mother in these situations could not be analyzed. Second, this investigation analyzed a simulation of a vaginal delivery, but the procedures and conditions of a cesarean section or other types of labor could vary these results. Third, having the mother in the room during resuscitation could be beneficial, but her reaction in this situation could not be predicted, as well as how this situation could affect her mental health. For this reason, future studies should consider this point when analyzing RCP with skin-to-skin contact. Future research should also consider bigger sample sizes and the different types of labor in order to improve the understanding of these findings. Furthermore, the predisposition of health care professionals to conduct SSC-CPR in a real situation is still unknown. A recent investigation reported that between 24% and 35% of newborn resuscitators thought that performing CPR on a table near the mother had some adverse impact on emergency care [41]. For this reason, future research should also consider increasing the level of training of professionals in SSC-CPR in order to improve the execution of the maneuvers [42]. Moreover, training the professionals of maternity units in SSC-CPR could have a real impact on mothers and infants.

## 5. Conclusions

In conclusion, SSC-CPR performed by trained rescuers appears to be feasible and able to be performed with high quality. Performing CPR on a newborn while maintaining skin-to-skin contact with the mother appears not to decrease the quality of the maneuver and may have positive effects on the mother and the newborn.

## Figures and Tables

**Table 1:** Results of compression variables.

	Std-CPR		SSC-CPR		*p*-Value (ES)
	Me	IQR	Me	RIC	
Total number of CCs	160	133–177	147	130–169	0.06
Mean rate (CCs/min)	136	126–149	131	119–142	0.004 (0.46)
Mean depth (mm)	34	31–37	34	29–38	0.66
CCs with adequate rate (%)	59	11–89	77	40–94	0.06
CCs with adequate depth (%)	18	3–38	19	4–36	0.57
CCs with adequate release (%)	99	95–100	100	97–100	0.07
CCs with adequate hand-position (%)	100	96–100	100	100–100	0.06

Me: median; IQR: interquartile range; Std-CPR: standard cardiopulmonary resuscitation; SSC-CPR: cardiopulmonary resuscitation with skin-to-skin contact; CCs: chest compressions; ES: effect size.

**Table 2:** Results of ventilation variables.

	Std-CPR		SSC-CPR		*p*-Value (ES)
	Me	IQR	Me	RIC	
Number of total Vs	58	49–64	54	48–61	0.06
Effective Vs (%)	100	88–100	100	98–100	0.40
Mean volume (mL)	23	20–26	21	17–25	0.042 (0.45)

Me: median; IQR: interquartile range; Std-CPR: standard cardiopulmonary resuscitation; SSC-CPR: cardiopulmonary resuscitation with skin-to-skin contact; Vs: ventilations, ES: effect size.

**Table 3:** Results of quality variables.

	Std-CPR		SSC-CPR		*p*-Value
	Me	IQR	Me	IQR	
CC quality (%)	74	53–89	74	58–87	0.79
V quality (%)	94	84–97	96	93–99	0.12
CPR quality (%)	75	55–87	82	70–91	0.06

Me: median; IQR: interquartile range; Std-CPR: standard cardiopulmonary resuscitation; SSC-CPR: cardiopulmonary resuscitation with skin-to-skin contact.

## Data Availability

No new data were created or analyzed in this study. Data sharing is not applicable to this article.
